# Apparent propagator anisotropy from single‐shell diffusion MRI acquisitions

**DOI:** 10.1002/mrm.28620

**Published:** 2020-12-13

**Authors:** Santiago Aja‐Fernández, Antonio Tristán‐Vega, Derek K. Jones

**Affiliations:** ^1^ Laboratorio de Procesado de Imagen Universidad de Valladolid Valladolid Spain; ^2^ Cardiff University Brain Research Imaging Centre (CUBRIC), School of Psychology Cardiff University Cardiff UK

**Keywords:** diffusion MRI, EAP, HARDI, microstructure, propagator anisotropy

## Abstract

**Purpose:**

The apparent propagator anisotropy (APA) is a new diffusion MRI metric that, while drawing on the benefits of the ensemble averaged propagator anisotropy (PA) compared to the fractional anisotropy (FA), can be estimated from single‐shell data.

**Theory and Methods:**

Computation of the full PA requires acquisition of large datasets with many diffusion directions and different b‐values, and results in extremely long processing times. This has hindered adoption of the PA by the community, despite evidence that it provides meaningful information beyond the FA. Calculation of the complete propagator can be avoided under the hypothesis that a similar sensitivity/specificity may be achieved from *apparent* measurements at a given shell. Assuming that diffusion anisotropy (DiA) is nondependent on the b‐value, a closed‐form expression using information from one single shell (ie, b‐value) is reported.

**Results:**

Publicly available databases with healthy and diseased subjects are used to compare the APA against other anisotropy measures. The structural information provided by the APA correlates with that provided by the PA for healthy subjects, while it also reveals statistically relevant differences in white matter regions for two pathologies, with a higher reliability than the FA. Additionally, APA has a computational complexity similar to the FA, with processing‐times several orders of magnitude below the PA.

**Conclusions:**

The APA can extract more relevant white matter information than the FA, without any additional demands on data acquisition. This makes APA an attractive option for adoption into existing diffusion MRI analysis pipelines.

## 1 INTRODUCTION

The term diffusion magnetic resonance imaging (dMRI) refers to a set of diverse imaging techniques that, when applied to brain studies, provide useful information about the microscopic organization and connectivity of the white matter. One relevant feature of dMRI is its ability to measure orientational variance in the different tissues, that is, anisotropy. Nowadays, the most common way to estimate the anisotropy is via the diffusion tensor (DT).^1^ Diffusion tensor MRI (DT‐MRI) brought to light one of the common issues of dMRI techniques: in order to carry out clinical studies, the information given by the selected diffusion analysis method must be translated into some scalar measures that describe different features of diffusion within every voxel. That way, metrics like the fractional anisotropy (FA) were defined with the DT as a starting point.^1^ Despite the strong limitations that the underlying Gaussian assumption imposes, the FA is still widely used in clinical studies involving dMRI.

In practice, the diffusion mechanisms cannot be fully described by DT‐MRI because of the oversimplified Gaussian fitting. Accordingly, techniques with more degrees‐of‐freedom naturally arose, such as diffusion kurtosis imaging (DKI)^2^ or methods based on high angular resolution diffusion imaging (HARDI).^3^, ^4^ The trend over the last decade has been to acquire a large number of diffusion‐weighted images distributed over several shells (ie, with several gradient strengths) and with moderate‐to‐high b‐values to estimate more advanced diffusion descriptors, such as the ensemble average diffusion propagator (EAP).^5^ The estimation relies on model‐free, nonparametric approaches that can accurately describe most of the relevant diffusion phenomena.

The most straightforward strategy to estimate the EAP is to sample the Cartesian q‐space densely enabling diffusion spectrum imaging (DSI),^6^ which requires a vast number of acquisitions. Alternatively, several methods were proposed grounded on sparse samplings of the q‐space, being the most prominent: hybrid diffusion imaging (HYDI),^7^, ^8^ multiple q‐shell diffusion propagator imaging (mq‐DPI),^9^, ^10^ Bessel Fourier orientation reconstruction (BFOR),^11^ the directional Radial Basis Functions (RBFs),^12^ the mean apparent propagator MRI (MAP‐MRI),^5^, ^13^ or the Laplacian‐regularized MAP‐MRI (MAPL).^14^

Regardless of the method selected for estimating the EAP, the typical end‐user condenses the information provided by the whole EAP into a set of scalar metrics such as: the probability of zero displacement (or return‐to‐origin probability, RTOP), the q‐space inverse variance, the return‐to‐plane (RTPP) and return‐to‐axis probabilities (RTAP),^8^, ^12^, ^15^ or the propagator anisotropy (PA).^5^ In this work, we will focus on the latter.

The PA can be seen as an alternative anisotropy measure able to discern changes that remain hidden for the FA. It reveals microstructural information of interest in the white matter. For example, a recent study in transgenic rats suggests that the PA may be a valid biomarker for Alzheimer’s disease.^16^ The same study also shows that the PA could be an important marker in longitudinal studies, indicating a possible dependency with age. Ref. [13] showed that the PA shows higher tissue contrast than the FA in white matter. Finally, in Ref. [17], the main limitation of the PA was detected: the bottleneck of studies with EAP‐derived measures is the amount of data needed for the calculation. This issue, together with the long processing times needed for EAP imaging, has slowed down a widespread adoption of propagator‐based anisotropy measures by the clinical community and motivated the current work.

This same pitfall has been recently addressed in Ref. [18] for the computation of other EAP imaging‐related markers (namely RTOP, RTPP, and RTAP). The so‐called “Apparent Measures Using Reduced Acquisitions” (AMURA) can mimic the sensitivity of EAP‐based measures to microstructural changes when a reduced amount of data distributed in a few shells (even one) is available. AMURA assumes a prior model for the behavior of the radial q‐space instead of trying to numerically describe it, yielding closed‐form expressions that can be computed easily even from single‐shell acquisitions.

The present paper extends AMURA to the estimation of the PA. To that end, the same constrained model for radial diffusion used in Ref. [18] is adopted here, that is, the DiA is assumed to be independent of the actual b‐value of the measured shells. We use this simplification to derive alternative closed‐forms for the inner products that define the original PA that can be computed even from single‐shell acquisitions. At the same time, the so‐called apparent propagator anisotropy (APA), together with other closely related measures we derive from it, may reveal analogous tissue anisotropy features as the original PA and other anisotropy measures. The use of a constrained model, instead of regularizing a heavily under‐determined problem, makes the APA more robust for certain brain structures than the PA itself, as we illustrate over an extensive set of experiments performed on data acquired with a ’clinical’ type acquisition.

## 2 THEORY

### 2.1 The diffusion signal

The EAP, *P*(**R**), is the probability density function of the water molecules inside a voxel moving an effective distance **R** in a time Δ. It is related to the normalized magnitude signal provided by the MRI scanner, *E*(**q**), by the Fourier transform F{.} ^19^:

(1) $$P\left(R\right)\;=\;\int_{R^{3}}E\left(q\right)e^{-2\pi jq\cdot R}dq\;=\;F\left\{|E(q)|\right\}\left(R\right).$$

The inference of exact information on the **R**‐space would require the sampling of the whole **q**‐space to exploit the Fourier relationship between both spaces.

In order to obtain a closed‐form analytical representation from a reduced number of acquired images, a model of the diffusion behavior must be adopted. The most common techniques rely on the assumption of a Gaussian diffusion profile and a steady state regime of the diffusion process leading to DT representation.^20^ Alternatively, a more general expression for *E*(**q**) can be used^21^:

(2) $$E\left(q\right)\;=\;\exp\left(‐4\pi^{2}\tau q_{0}^{2}D\left(q\right)\right)\;=\;\exp\left(‐b\cdot D(q)\right)$$

where the positive function $D\left(q\right)\;=\;D\left(q_{0},\;\theta ,\;\phi \right)$ is the apparent diffusion coefficient (ADC), $b\;=\;4\pi^{2}\tau {\left\|q\right\|}^{2}$ is the so‐called b‐value, $q_{0}\;=\;\left\|q\right\|$ and *θ*, *ϕ* are the angular coordinates in the spherical system. The effective diffusion time *τ* is defined as *τ* = Δ−*δ*/3, where the diffusion time Δ is usually corrected with the pulse duration *δ*.

The monoexponential assumption is ubiquitous to many HARDI techniques, and it implies the anisotropy of the diffusion signal is roughly independent of the b‐value. The accuracy of such an assumption depends on the range of b‐values considered: according to Ref. [21], this monoexponential signal representation is predominant in the mammalian brain for b‐values up to 2000 $s/{\mathrm{mm}}^{2}$. Beyond this value, in the range 2000‐10,000 $s/{\mathrm{mm}}^{2}$, it has been proven that the deviation of the actual signal from monoexponentials embeds meaningful information about the diffusion process.^5^ However, the relevance of this extra information might be at stake due to the limitations inherent to commonly used samplings (with maximum b‐values ranging 3000 to 5000 $s/{\mathrm{mm}}^{2}$), which are able to capture only the low‐frequency spectrum.

### 2.2 Propagator anisotropy and inner product

In Ref. [5], the authors propose a measure called the PA that quantifies how the propagator diverges from the isotropic one. The PA is defined as a function of the sine of the angle between two propagators as:

(3) $$\text{PA}\;=\;\gamma \left(\sin\left(∠[P\left(R\right),\;P_{I}\left(R\right)]\right),\;\epsilon \right),$$

where *P*(**R**) is the actual propagator and $P_{I}\left(R\right)$ its equivalent isotropic propagator. The function *γ*(.,*ε*) is a contrast enhancement to better distribute the output values in the range [0, 1]. For the sake of simplicity, hereon, we will use $\theta_{P,P_{I}}$ to denote the angle.

In order to calculate this metric, we need to define the inner product between two propagators. Let $P_{1}\left(R\right)$ and $P_{2}\left(R\right)$ be two different propagators. If we consider them as two different signals defined over a common signal space S, we can define an inner product as^5^, ^22^:

(4) $$〈P_{1}\left(R\right),\;P_{2}\left(R\right)〉=\int_{R^{3}}P_{1}\left(R\right)P_{2}^{*}\left(R\right)dR.$$

where $P_{2}^{*}\left(R\right)$ is the conjugate of $P_{2}\left(R\right)$. According to Parseval’s Theorem,^22^ since variables **R** and **q** are related via the Fourier transform, there is an equivalence of this product in the **q**‐space. Considering that the magnitude‐reconstructed diffusion‐weighted MR signal *E*(**q**) is always real and symmetric, $E^{*}\left(q\right)\;=\;E\left(q\right)$ and *E*(**q**) = *E*(−**q**), we can write:

(5) $$〈P_{1}\left(R\right),\;P_{2}\left(R\right)〉=\int_{R^{3}}E_{1}\left(q\right)E_{2}\left(q\right)dq,$$

where $E_{1}\left(q\right)=F^{‐1}\left\{P_{1}\left(R\right)\right\}\left(q\right)$ and $E_{2}\left(q\right)\;=\;F^{‐1}\left\{P_{2}\left(R\right)\right\}\left(q\right)$. The norm of a signal is defined as:

(6) $$||P_{1}\left(R\right)||={〈P_{1}\left(R\right),P_{1}\left(R\right)〉}^{1/2}={\left(\int_{R^{3}}{\left|E_{1}\left(q\right)\right|}^{2}dq\right)}^{1/2}$$

The *similarity* between two signals is given by the cosine of the angle between them, defined as:

(7) $$\cos\theta_{P_{1},P_{2}}\;=\;\frac{\left\langle P_{1}\left(R\right),\;P_{2}\left(R\right)\right\rangle }{\left\|P_{1}\left(R\right)\|\cdot \|P_{2}\left(R\right)\right\|}.$$

The sine is calculated from Equation (7) as:

(8) $$\sin\theta_{P_{1},P_{2}}=\sqrt{1‐\cos^{2}\theta_{P_{1},P_{2}}.}$$

This result can be extrapolated for the EAP, *P*(**R**), and its isotropic equivalent, $P_{1}\left(R\right)$, to define the PA as in Equation (3).

## 3 METHODS

### 3.1 Apparent propagator anisotropy

The calculation of the PA demands the full estimation of the EAP which requires an extensive data acquisition. In contrast, AMURA permits the use single‐shell data at the expense of constraining the radial behavior so that the diffusivity *D*(**q**) does not depend on the radial direction (ie, independent of the magnitude of the q‐vector): *D*(**q**) = *D*(**u**), where ‖**u**‖ = 1 and **q** = *q* **u**.^18^ Then, Equation (2) becomes:

(9) $$E\left(q\right)\;=\;E\left(q,\;u\right)\;=\;\exp\left(‐4\pi^{2}\tau q^{2}\;D\left(u\right)\right).$$

Note that, although *D*(**q**) is independent of *q*, the signal attenuation, *E*(**q**), still has *q*‐dependence. This assumption, although restrictive, is used to define certain diffusion representations in HARDI,^4^, ^23^ where only one data shell (ie, b‐value) is usually acquired.

In what follows, we explicitly calculate the inner product that defines the PA by using the simplification in Equation (9), yielding an anisotropy metric related to the PA for a specific shell. *First*, we define an isotropic signal equivalent to the monoexponential model, $E_{I}\left(q\right)$. Pursuing an analogous formulation to that in AMURA,^18^ we propose an alternative formulation, leading to a linear computation:

(10) $$E_{I}\left(q\right)\overset{\Delta }{\;=\;}\exp\left(‐4\pi^{2}\tau q^{2}\;D_{\mathrm{AV}}\right),$$

for:

(11) $$D_{\mathrm{AV}}\;=\;\frac{1}{4\pi }\int_{S}D\left(u\right)du.$$

The integration on the surface of the sphere from a limited number of samples is performed by fitting corresponding signals in the basis of spherical harmonics (SH), whose 0th order coefficient is defined as:

(12) $$C_{0,0}\left\{H(u)\right\}\;=\;\frac{1}{\sqrt{4\pi }}\int_{S}H\left(u\right)du.$$

Therefore, $D_{\mathrm{AV}}$ can be calculated as:

(13) $$D_{\mathrm{AV}}\;=\;\frac{1}{\sqrt{4\pi }}C_{0,0}\left\{D(u)\right\},\mathrm{so}\;\mathrm{that}\;\;E_{I}\left(q\right)\;=\;\exp\left(‐2\pi^{3/2}\tau q^{2}\;C_{0,0}\left\{D(u)\right\}\right).$$

*Second*, we calculate the norm of *P*(**R**) and $P_{I}\left(R\right)$ under the considered assumption:

(14) $$\begin{array}{cc} {\left||P\left(R\right)|\right|}^{2} & =\int_{R^{3}}\exp\left(‐4\pi^{2}\tau q^{2}\;2\;D\left(u\right)\right)dq\\ & =\int_{0}^{\infty }\int_{S}\exp\left(‐4\pi^{2}\tau q^{2}\;2\;D\left(u\right)\right)q^{2}du\;dq\\ & =C_{p}\int_{S}\frac{1}{{\left(2\cdot D\left(u\right)\right)}^{3/2}}du \end{array}$$

(15) $$=C_{p}\cdot \sqrt{\frac{\pi }{2}}\cdot C_{0,0}\left\{D{\left(u\right)}^{‐3/2}\right\},$$

where $C_{p}$ is a constant. Following the same reasoning, the norm of the isotropic equivalent is:

(16) $$\begin{array}{c} \left|\right|P_{1}\left(R\right){\left|\right|}^{2}=\int_{R^{3}}\exp\left(‐4\pi^{2}\tau q^{2}2D_{\mathrm{AV}}\right)dq\\ \;\;\;\;\;\;\;\;\;\;\;\;\;\;\;\;\;\;\;\;\;=\;C_{p}\sqrt{2\pi }\cdot D_{\mathrm{AV}}^{‐3/2}. \end{array}$$

*Third*, we calculate the inner product of both signals using the single‐shell assumption:

(17) $$\begin{array}{cc} \left\langle P\left(R\right),P_{I}\left(R\right)\right\rangle & =\int_{R^{3}}\exp\left(‐4\pi^{2}\tau q^{2}\;\left(D\left(u\right)+D_{\mathrm{AV}}\right)\right)dq\\ & =C_{p}\int_{S}\frac{1}{{\left(D\left(u\right)+D_{\mathrm{AV}}\right)}^{3/2}}dS \end{array}$$

(18) $$=C_{p}\cdot \sqrt{4\pi }\cdot C_{0,0}\left\{{\left(D\left(u\right)+D_{\mathrm{AV}}\right)}^{‐3/2}\right\}.$$

*Next*, we calculate the cosine and sine of the angle between both signals:

(19) $$\begin{array}{cc} \cos^{2}\theta_{P,P_{I}} & =\frac{{\left\langle P\left(R\right),\;P_{I}\left(R\right)\right\rangle }^{2}}{{\left||P\left(R\right)|\right|}^{2}\cdot \left|\right|P_{I}\left(R\right){\left|\right|}^{2}}\\ & =\frac{4}{\sqrt{\pi }}\frac{{\left[C_{0,0}\left\{{\left(D\left(u\right)+D_{\mathrm{AV}}\right)}^{‐3/2}\right\}\right]}^{2}}{C_{0,0}\left\{\cdot D{\left(u\right)}^{‐3/2}\right\}\cdot D_{\mathrm{AV}}^{‐3/2}}; \end{array}$$

(20) $$\sin\theta_{P,P_{I}}=\sqrt{1‐\cos^{2}\theta_{P,P_{I}}}.$$

From here, we can define the anisotropy measure prior to the nonlinear transformation as:

(21) $${\text{APA}}_{0}\;=\;\sin\theta_{P,P_{I}}.$$

*Finally*, the PA is calculated using the Gamma transformation proposed by Ref. [5]:

(22) $$\gamma \left(t,\;\epsilon \right)\;=\;\frac{t^{3\epsilon }}{1‐3t^{\epsilon }+3t^{2\epsilon }}.$$

This way, the APA at a given b‐value is calculated as:

(23) $$\text{APA}\;=\;\gamma (\sin\theta_{P,P_{I}},\;\epsilon ).$$

### 3.2 Alternative form of the APA

The need for a contrast enhancement of the raw values of the PA through the gamma correction in Equation (23) was already recognized by Ref. [5]. Generalizing this idea, we can apply a contrast enhancement to the attenuation signal itself before the PA is actually computed. Since *E*(**q**) is bounded in the range (0, 1), the negative logarithm of *E*(**q**), that is, *D*(**q**), is an appropriate transformation in this sense. Hence, we can reformulate:

(24) $$\left\langle D_{1}\left(q\right),\;D_{2}\left(q\right)\right\rangle \;=\;\int_{S}D_{1}\left(u\right)D_{2}\left(u\right)du;$$

(25) $${\left||D\left(q\right)|\right|}^{2}\;=\;\int_{S}D^{2}\left(u\right)du,$$

and the DiA is defined straightforwardly as:

(26) $$\begin{array}{c} \mathrm{DiA}={\sin\theta }_{D,D_{\mathrm{AV}}}\\ \;\;\;\;\;\;\;\;\;\;=\sqrt{1‐\frac{{\left[D_{\mathrm{AV}}\cdot \int_{S}D\left(u\right)du\right]}^{2}}{4\pi \cdot D_{\mathrm{AV}}^{2}\cdot \int_{S}D^{2}\left(u\right)du}}\\ \;\;\;\;\;\;\;\;\;\;\;={\left(\frac{C_{0,0}\left\{D^{2}\left(u\right)\right\}‐\frac{1}{\sqrt{4\pi }}\cdot C_{0,0}^{2}\left\{D\left(u\right)\right\}}{C_{0,0}\left\{D^{2}\left(u\right)\right\}}\right)}^{1/2}. \end{array}$$

so that the term $D_{\mathrm{AV}}$ no longer appears. The DiA can be seen as a generalization of the coefficient of variation of the diffusion (CVD) defined in Ref. [24] as a robust alternative for the FA. According to Ref. [25], the DiA is also an alternative definition to the generalized anisotropy proposed by Ref. [26]. Note that the derived DiA also resembles to the generalized fractional anisotropy (GFA) defined in Ref. [27].

An overview of all the proposed diffusion anisotropy metrics, together with their specific numerical implementations, is presented in Table 1.

**TABLE 1:** Summary of the proposed anisotropic diffusion metrics

Measure	Formula	Practical implementation
APA	$\gamma \left({\text{APA}}_{0},\epsilon \right)$	$\gamma \left({\text{APA}}_{0},\epsilon \right)$
${\mathrm{APA}}_{0}$	$\sqrt{1‐\frac{{\left[\int_{S}{\left(D\left(u\right)+D_{\mathrm{AV}}\right)}^{‐3/2}du\right]}^{2}}{\sqrt{2}\pi D_{\mathrm{AV}}^{‐3/2}\int_{S}{\left(2D\left(u\right)\right)}^{‐3/2}du}}$	$\sqrt{1‐\frac{4}{\sqrt{\pi }}\frac{{\left[C_{0,0}\left\{{\left(D\left(u\right)+D_{\mathrm{AV}}\right)}^{‐3/2}\right\}\right]}^{2}}{C_{0,0}\left\{D{\left(u\right)}^{‐3/2}\right\}\cdot D_{\mathrm{AV}}^{‐3/2}}}$
$D_{\mathrm{AV}}$	$\frac{1}{4\pi }\int_{S}D\left(u\right)du$	$\frac{1}{\sqrt{4\pi }}C_{0,0}\left\{D(u)\right\}$
DiA	$\sqrt{\frac{4\pi \cdot \int_{S}D^{2}\left(u\right)du‐{\left[\int_{S}D\left(u\right)du\right]}^{2}}{4\pi \cdot \int_{S}D^{2}\left(u\right)du}}$	$\sqrt{1‐\frac{\frac{1}{\sqrt{4\pi }}\cdot C_{0,0}^{2}\left\{D\left(u\right)\right\}}{C_{0,0}\left\{D^{2}\left(u\right)\right\}}}$

### 3.3 Public datasets used for the experiments

In order to test the proposed measures for a wide range of acquisition protocols and MR hardware configurations, four different datasets were used:

1. *Human Connectome Project (HCP)* (Data obtained from the Human Connectome Project (HCP) database (ida.loni.usc.edu/login.jsp). The HCP project (Principal Investigators: Bruce Rosen, M.D., Ph.D., Martinos Center at Massachusetts General Hospital; Arthur W. Toga, Ph.D., University of Southern California, Van J. Weeden, MD, Martinos Center at Massachusetts General Hospital) is supported by the National Institute of Dental and Craniofacial Research (NIDCR), the National Institute of Mental Health (NIMH) and the National Institute of Neurological Disorders and Stroke (NINDS). HCP is the result of efforts of co‐investigators from the University of Southern California, Martinos Center for Biomedical Imaging at Massachusetts General Hospital (MGH), Washington University, and the University of Minnesota.): specifically volumes MGH1007, acquired on a Siemens 3T Connectom scanner with four different shells at $b\;=\;[1000,\;3000,\;5000,\;10,000]\;s/{\mathrm{mm}}^{2}$, with [64, 64, 128, 256] gradient directions each, in‐plane resolution 1.5 mm and slice thickness was 1.5 mm.
2. *Public Parkinson’s disease database (PPD)*: publicly available database (www.nitrc.org/frs/?group\_id=835) acquired in the Cyclotron Research Centre, University of Liège. It consists of 53 subjects in a cross‐sectional Parkinson’s disease (PD) study: 27 PD patients and 26 age, sex, and education‐matched control subjects. Data were acquired on a 3T head‐only MR scanner (Magnetom Allegra, Siemens Medical Solutions, Erlangen, Germany) operated with an eight‐channel head coil. DWIs were acquired with a twice‐refocused spin‐echo sequence with EPI readout at two shells $b\;=\;[1000,\;2500,\;5000,\;10,000]\;s/{\mathrm{mm}}^{2}$ along 120 encoding gradients. Acquisition parameters are TR = 6800 ms, TE = 91 ms, and FOV = 211 ${\mathrm{mm}}^{2}$, voxel size 2.4 × 2.4 × 2.4 mm, no parallel imaging and 6/8 partial Fourier were used. More information can be found in Ref. [28].
3. *ADNI database (ADNI)*: multi‐shell data from 55 subjects were obtained from the Alzheimer’s Disease Neuroimaging Initiative (ADNI) database (Data used in preparation of this article were obtained from ADNI database (adni.loni.isc.edu). As such, the investigators within the ADNI contributed to the design and implementation of ADNI and/or provided data but did not participate in analysis or writing of this report. A complete listing of ADNI investigators can be found at: http://adni.loni.usc.edu/wp‐content/uploads/how_to_apply/ADNI_Acknowledgement_List.pdf. The ADNI was launched in 2003 as a public‐private partnership, led by Principal Investigator Michael W. Weiner, MD. The primary goal of ADNI has been to test whether serial MRI, PET, other biological markers, and clinical and neuropsychological assessment can be combined to measure the progression of MCI and early Alzheimer’s disease (AD). From the whole database, we have focused on those subjects scanned with more than one shell (ADNI 3 advanced protocol). The data used consist of 38 cognitively normal elderly controls (CN; mean age: 71.4 ± 6.4 years, 15 M/23 F) and 17 with mild cognitive impairment (MCI; mean age: 71.6 ± 8.6 years, 10 M/7 F). Data were acquired on 3T Siemens Advanced Prisma scanners (at 9 different acquisition sites). DW images were acquired at three distinct b‐values $b\;=\;\left[500,\;1000,\;2000,\;10,000\right]\;s/{\mathrm{mm}}^{2}$ with different encoding gradients for each shell: 6 ($b\;=\;500\;s/{\mathrm{mm}}^{2}$), 48 ($b\;=\;1000\;s/{\mathrm{mm}}^{2}$), 60 ($b\;=\;2000\;s/{\mathrm{mm}}^{2}$) and 12 unweighted (b  =  0) volumes. Acquisition parameters are TR = 3300 ms, TE = 71, 116 × 116 matrix, 81 slices, voxel size 2 × 2 × 2 mm, whole scanned volume 232 × 232 × 160 mm. All raw DWI were corrected for motion, eddy‐current and echo‐planar imaging (EPI) induced susceptibility artifacts and B1 field inhomogeneity.
4. *Multi‐shell data acquired at CUBRIC (CBR* www.cardiff.ac.uk/cardiff‐university‐brain‐research‐imaging‐centre/research/projects/cross‐scanner‐and‐cross‐protocol‐diffusion‐MRI‐data‐harmonisation): 14 healthy volunteers scanned on a 3T Siemens Prisma scanner (80 mT/m) with a pulsed‐gradient spin‐echo (PGSE) sequence. Three shells were acquired at $b\;=\;[1200,\;3000,\;5000]\;s/{\mathrm{mm}}^{2}$ with 60 directions per value. The resolution is 1.5 × 1.5 × 1.5 mm. Other acquisition parameters are: TE = 80 ms, TR = 4500 ms, Δ/*δ* = 38.3/19.5 ms, parallel imaging acquisition (GRAPPA2) with sum of squares combination and 32 channels.

## 4 RESULTS

### 4.1 Visual assessment

**FIGURE 1:**
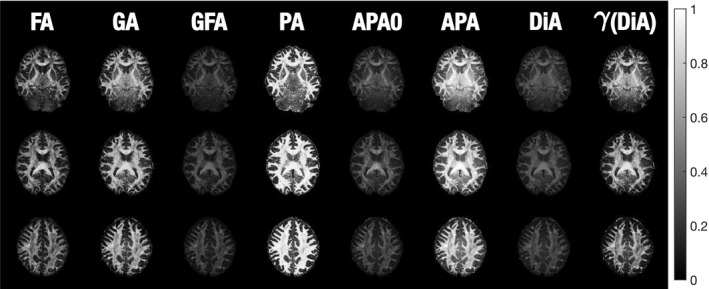
Visual comparison of the diffusion anisotropy metrics using slices 42, 52, and 65 of the MGH1007 volume from HCP. FA is calculated using $b\;=\;1000\;s/{\mathrm{mm}}^{2}$, GA, GFA, APA0, APA, and DiA using $b\;=\;3000\;s/{\mathrm{mm}}^{2}$, and PA using 4 shells (1000, 3000, 5000, and 10,000 $s/{\mathrm{mm}}^{2}$). *γ*(DiA) is the gamma‐corrected version of DiA, constructed for visualization purposes

A preliminary visual assessment of the different metrics was performed using three slices (42, 52, and 65) from the HCP volume MGH1007. The proposed measures (${\mathrm{APA}}_{0}$, APA, and DiA) were calculated using a single shell at $b\;=\;3000\;s/{\mathrm{mm}}^{2}$. For the sake of comparison, we have also calculated the FA at $b\;=\;1000\;s/{\mathrm{mm}}^{2}$, the GA and GFA (calculated using FSL) at $b\;=\;3000\;s/{\mathrm{mm}}^{2}$, and the PA using all the available information (four shells). Results are shown in Figure 1. A gamma‐corrected version of DiA is also presented to enhance the contrast. It is calculated using the transformation in Equation (22) over Equation (26). As expected, all the metrics highlight the anisotropy of the white matter, meanwhile they suppress the signal from the (approximately) isotropic gray matter. isotropic gray matter. ${\mathrm{APA}}_{0}$ and DiA are not uniformly distributed over the range [0, 1], an effect also present in the GFA, which is palliated by the APA. Comparing the new measures with the original PA, the latter seems over‐saturated toward 1, in a way that most of the white matter looks homogeneous. Conversely, the APA exhibits wider dynamic range across the white matter, making it possible to distinguish different anatomical features.

**FIGURE 2:**
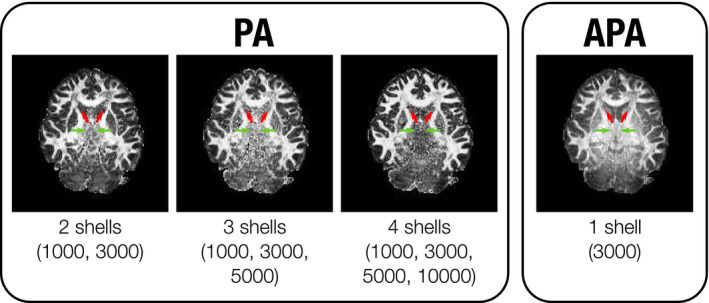
Visual comparison of PA to APA using slice 42 of the MGH1007 volume from HCP. APA is calculated using one single shell ($b\;=\;3000\;s/{\mathrm{mm}}^{2}$), and PA using two (1000 and $3000\;s/{\mathrm{mm}}^{2}$), three (1000, 3000, and $5000\;s/{\mathrm{mm}}^{2}$) and four shells (1000, 3000, 5000, and 10,000 $s/{\mathrm{mm}}^{2}$). There are marked differences between APA and PA in the basal ganglia, including the head of caudate (red arrows) and thalamus (green arrows)

Moreover, Figure 2 suggests that the APA exhibits a good noise behavior across the entire cerebrum, even in those areas with low anisotropy such as the CSF (which has low APA) and areas of intermediate anisotropy, such as thalamus and head of caudate. This is in stark contrast to the PA computed using MAP‐MRI, where there is elevated anisotropy in the ventricles, and the poor contrast‐to‐noise ratio in the basal ganglia occludes corresponding structures.

### 4.2 Validation with clinical data

The next set of experiments aims at quantitatively evaluating the potential of the new metrics for the clinical analysis of real data provided in public databases. The assessment is based on the ability to find significant differences between two different cases: (a) mild cognitive impairment (MCI), using the ADNI database, and (b) Parkinson disease (PD), using the PPD database. We have selected these two cases as they are illustrative of very different clinical studies: according to the literature, significant differences in diffusion anisotropy can be easily found between MCI and controls in a large number of brain regions.^29^, ^30^ In contrast, although patient‐control anisotropy differences have been reported in white matter regions for PD,^31^ such differences are harder to find using standard dMRI analysis. This way, the ability of the new measures to detect pathology is evaluated under two different difficulty levels.

For all the experiments, the FA was calculated as a reference value using MRTRIX^32^ (mrtrix.org) from the data collected at $b\;=\;1000\;s/{\mathrm{mm}}^{2}$. The FA maps of all the volumes were warped to a common template using the standard TBSS pipeline.^33^ The same transformation was applied to all the metrics considered for the experiment.

Let us focus first on the *MCI experiment*. The FA was compared to the APA using two different shells for both measures (*b* = 1000 and $b\;=\;2000\;s/{\mathrm{mm}}^{2}$) in order to check the capability of the latter to discriminate differences between MCI and healthy controls. To that end, a region of interest (ROI) analysis was carried out: 48 different ROI were identified on the subjects using the JHU WM atlas.^34^ For the sake of robustness, only those 22 ROIs containing more than 2500 voxels were considered for the experiment. The average value of the FA and the APA inside each ROI was calculated using the 2% and 98% percentiles. Then we carried out a two‐sample, pooled variance *t*‐test between controls and patients for each of the measures considered and at each of the 22 ROIs. To observe the dependence of the measures with the number of subjects, the *t*‐test was repeated in subsamples of the original set. Starting with 55 subjects (38 CN and 17 MCI), the number of subjects per group was progressively reduced in 3 subjects (2 CN and 1 MCI) for each iteration, until no regions with significant differences were found. For each iteration, 200 repetitions were performed, each of them generating a random sub‐sample of subjects for which the inference was carried out. This inference plots differences between the two groups in a certain number of white matter regions with significance *P* < .05 (uncorrected). The median value of regions with significant differences across the 200 repetitions was considered as the figure of merit for each iteration.

**FIGURE 3:**
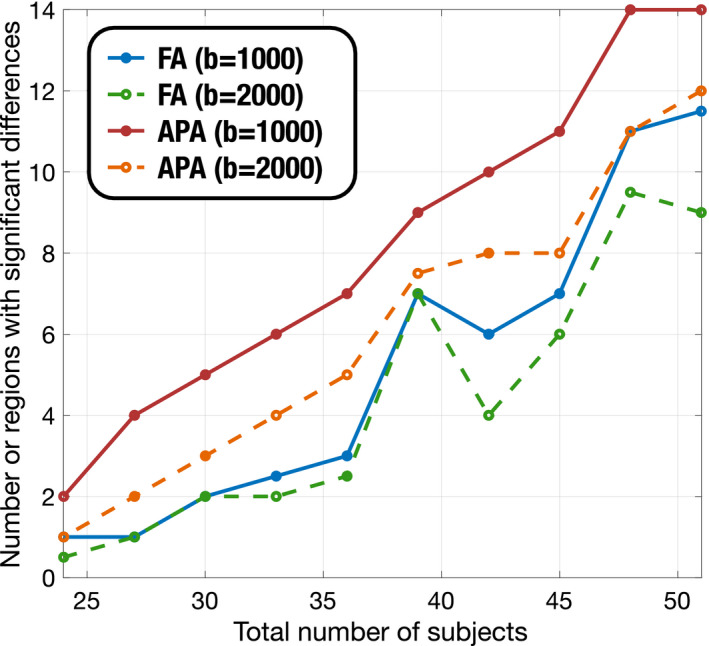
Number of regions with significant differences (*P* < .05) for different number of samples. FA is compared to APA for the ADNI database to find differences between control and MCI subjects. Only those regions with more than 2500 voxels are considered

Results are shown in Figure 3. As expected, the number of regions showing significant group differences decreased together with the number of subjects in each group. However, for any given sample size, the APA consistently finds a larger number of regions with significant patient‐control differences than the other metrics. Moreover, the APA is able to obtain similar results as the FA with a smaller sample size. This feature makes the APA a robust alternative to the FA even with datasets collected for DT‐MRI‐based analysis, that is, single‐shell data with $b\approx 1000\;s/{\mathrm{mm}}^{2}$. In this experiment, it is precisely at $b\;=\;1000\;s/{\mathrm{mm}}^{2}$ where the best discrimination results were obtained for the APA compared to the FA.

Complementarily, in order to test the sensitivity of APA compared to FA, we have conducted a McNemar’s statistical test with the results provided by bootstrapping for FA and APA with $b\;=\;1000\;s/{\mathrm{mm}}^{2}$, for each subsample set and the 200 repetitions. This type of test is usually employed to assess sensitivity and specificity of two different tests on the same sample. To that end, we have tested the null hypothesis that APA and FA detect differences in the same regions, and three alternative hypotheses: (a) APA detects more regions than FA (APA‐not(FA)); (b) FA detects more regions than APA (FA‐not(APA)); and (c) FA and APA detect different number of regions (two‐sided). Results can be seen in Table 2 where we show the number of regions with *P* < .01 for each subsample. Note that, according to the results, APA is able to detect differences in regions not detected by the FA (high values in the row APA‐not(FA)), while most of the findings reported by FA are in areas also reported by APA (low values in the row FA‐not(APA)).

**TABLE 2:** Results of the McNemar’s test on the ADNI data: comparison of areas detected by APA and FA (*P* < .01)

Number of subjects	51	48	45	42	39	36	33	30	27	24
Two‐sided	11	17	16	13	15	13	10	11	12	7
APA‐not(FA)	10	15	15	14	15	15	11	11	12	9
FA‐not(APA)	1	2	1	0	1	0	0	0	0	0

**FIGURE 4:**
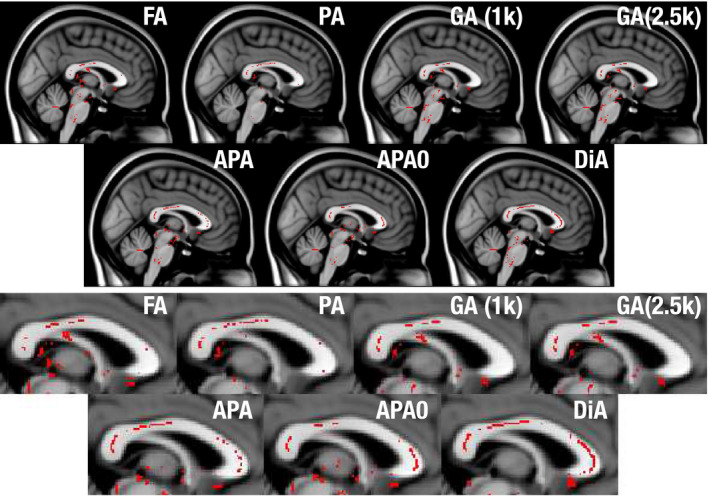
Significant differences found by statistical test for the Parkinson database, using a voxel‐wise analysis over the FA skeleton for the different considered metrics (sagittal view). In red, those points where the considered metric decreases in the PD with respect to the controls with statistical significance above 99% (*P* < .01)

**FIGURE 5:**
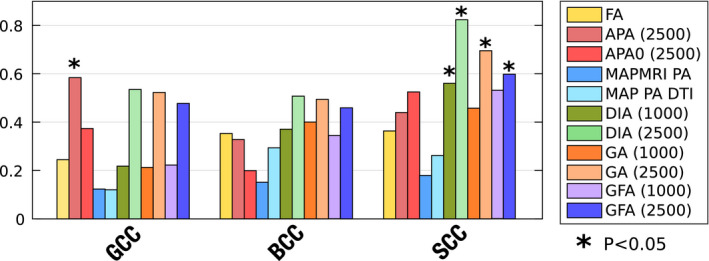
Absolute value of effect sizes (absolute Cohen’s d) for associations between PD and controls in the Parkinson data base for different metrics and, where appropriate, different b‐shells

Next, we test the utility of the new measures using the *PPD database*. Though PD is known to affect the substantia nigra or the gray matter more than the white matter, significant differences have also been reported in several white matter regions such as the corpus callosum (CC), the corticospinal tract and the fornix.^31^ The aim of this experiment was to test the ability of the proposed measures to detect differences in the white matter. Two different analysis were considered:

1. A voxelwise cross‐subject analysis using the FA skeleton with the randomise tool from the FSL toolbox (which performs a nonparametric permutation inference over the data) with 500 realizations. Those voxels with *P* < .01 (without TFCE) are highlighted in Figure 4. Voxels colored red denote where the considered metric decreases in the PD with respect to the controls.
2. A ROI oriented analysis: the three regions of the CC (genu—GCC, body—BCC, and splenium—SCC) were identified on the subjects using the JHU WM atlas.^34^ The average values of the different measures inside each ROI were calculated using the 2% and 98% percentiles. First, effect sizes were estimated using the Cohen’s *d*. Results are depicted in Figure 5. Then we carried out a two‐sample, pooled variance *t*‐test between controls and patients for each of the measures considered and at each of the three sections of the CC segmented in the JHU WM. Table 3 shows the results.

**TABLE 3:** Two‐sample, pooled variance, *t*‐tests for each measure and at each section of the corpus callosum: GCC (genu), BCC (body), and SCC (splenium)

	b‐value	GCC	BCC	SCC
FA	1000	0.378	0.205	0.192
MAPMRI‐PA	All	0.656	0.585	0.517
MAP‐PA‐DTI	All	0.664	0.290	0.345
GA	1000	0.443	0.151	0.102
	2500	0.063	0.078	0.015
GFA	1000	0.428	0.211	0.059
	2500	0.095	0.102	0.034
APA	1000	0.555	0.296	0.310
	2500	0.038	0.238	0.116
${\mathrm{APA}}_{0}$	1000	0.309	0.676	0.436
	2500	0.180	0.472	0.062
DIA	1000	0.431	0.183	0.047
	2500	0.057	0.071	0.004

*Notes*: The *P*‐values represent the probability that the averaged values (using the values between the 2% and 98% percentiles) of each region of the corresponding tract have identical means for both controls and patients. Differences with statistical significance above 99% are highlighted in green, and those with significance over 95% are highlighted in amber.

We have focused on the CC since this is the region where previous studies have reported group differences between PD and healthy controls. If we focus on this area in a sagittal plane in Figure 4, the FA and the GA only find some isolated voxels with statistically significant differences. The PA finds some extra voxels, but cannot show its true potential due to the small *b*‐values considered. In contrast, the proposed measures show more differences across the whole CC. All of them, especially the DiA, find differences in the genu of the CC (GCC). The slightly better performance of the DiA compared to the PA in this experiment supports the logarithmic contrast enhancement in the attenuation signal despite the uneven distribution of DiA values over the range [0, 1] seen in Figure 1.

In the ROI analysis, it is precisely at the SCC where all the measures show the greatest values of Cohen’s *d*, see Figure 5. Once again, DiA shows larger effect sizes, although the GA and GFA (with $b\;=\;2500\;s/{\mathrm{mm}}^{2}$) are also able to find significant differences in this ROI, see Table 3. However, note that the DiA shows a statistical significance above 99%. If we focus on the GCC ROI, only the APA is able to find differences. In contrast, the PA calculated with MAP‐MRI and the DTI version (proposed in Ref. [5]) both show very low effect sizes and are unable to detect significant differences in any part of the CC.

Finally, it is important to stress here that the aim of the experiments carried out in this section was not to demonstrate the clinical usefulness of APA in the particular case of MCI and PD, but rather to test its ability to detect differences in the white matter on real datasets. The fact that a particular measure finds significant patient‐control differences indicates that the diffusion properties it describes is altered by this particular pathology and/or in this particular dataset.

### 4.3 Sensitivity analysis to acquisition parameters

**FIGURE 6:**
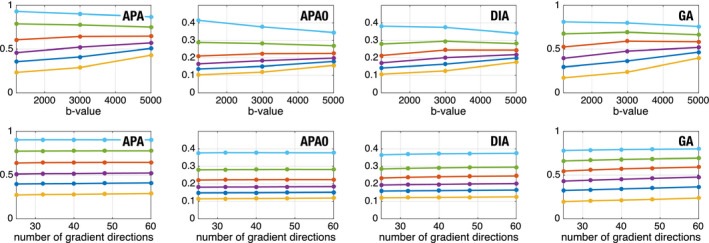
Evolution of the proposed measures with the b‐value (top) and the angular resolution (bottom), using data from a 3T Prisma scanner. The volume has been clustered in six different sets (for PA at $b\;=\;3000\;s/{\mathrm{mm}}^{2}$) and the median of each set is shown. Centroids of the data $C_{L}\;=\;\left\{0.27,\;0.41,\;0.52,\;0.65,\;0.78,\;0.91\right\}$

Next, we tested the dependency of APA on the b‐value and the number of diffusion samples taken in a given shell. To that end, we used five whole volumes from the CBR data. Each volume was divided in six different regions according to their diffusion features. The APA was first calculated and those voxels with APA < 0.1 removed. The remaining voxels were clustered in six different groups using k‐means (at $b\;=\;3000\;s/{\mathrm{mm}}^{2}$). Each voxel in the white matter was assigned to one cluster using its PA value and the minimum distance. The following test was carried out: first, the variability with the b‐value was probed by computing the different anisotropy measures with each of the available shells at $b\;=\;1200\;s/{\mathrm{mm}}^{2}$, $b\;=\;3000\;s/{\mathrm{mm}}^{2}$, or $b\;=\;5000\;s/{\mathrm{mm}}^{2}$. For the variability with the number of diffusion sampling directions, we began with the 60 samples at $b\;=\;3000\;s/{\mathrm{mm}}^{2}$ and uniformly downsampled this set to obtain either 25, 32, 40 and 48 diffusion directions subsets (A “uniform” downsampling of *n* gradients among the original 60 is here defined as those *n* directions that minimize the overall electrostatic repulsion energy among all ($\begin{array}{c} 60\\ n \end{array}$) combinations. The optimization is carried out using heuristic rules). All the proposed anisotropic diffusion measures were computed for each considered case, and the median value inside each of the six clusters is depicted in Figure 6.

Note that all the measures show a dependence on the b‐value: the smallest values tend to increase monotonically with the b‐value, whereas the higher values tend to show a monotonic decrease. However, and this is the key point, the separation between clusters remains the same for different b values. This means that the differences in the anisotropy detected by these measures can be detected when using different shells. All the measures show an extremely robust behavior to the variation in the number of sampling directions even in the case of very heavy downsampling.

### 4.4 Execution times

The long processing times associated with the estimation of EAP‐based measures is one of the issues that has hindered a widespread clinical adoption of the PA. In comparison, the linear nature of SH needed to estimate the APA results in a significant reduction of the calculation time, that can be several orders of magnitude faster than whole EAP‐based techniques.

To test this extreme, a volume from the PPD was used here to compute APA and PA measures on a quad‐core Intel(R) Core(TM) i7‐4770K 3.50GHz processor under Ubuntu Linux 16.04 SO. PA was calculated using the two available shells with MAP‐MRI using the DIPY library under Python 3.6.4 (scipy 1.0.0, the PA calculation is not available in the public distribution of DIPY. The current implementation has been kindly provided by Dr. Fick). APA was implemented using one single shell in MATLAB R2013b without multi‐threading. The calculation of APA took **3.17**s, while MAP‐MRI‐PA 2 h 53 min for the same volume. Though raw execution times are an ambiguous performance index (they can be dramatically improved, eg, via GPU acceleration), they give a reasonable idea of the relative complexity of each method. The calculation of the APA for the whole volume is almost instantaneous, which makes it feasible for practical studies.

## 5 DISCUSSION

The intention of the new anisotropy measure proposed here, APA, is not to exactly replicate a measure like the PA but, using a similar philosophy, to infer anatomical information with comparable discrimination power as the PA estimated using EAP‐based methods (mainly, MAP‐MRI). The original PA calculated from the EAP explicitly accounts for the radial behavior of the diffusion signal, which also needs to be sampled extensively. For the APA calculation, the radial behavior is not sampled but modeled as a monoexponential decay.

One might anticipate that the computation of the whole EAP would provide a more specific and sensitive measure than the APA, since the anisotropy information encoded in the radial direction is otherwise neglected in the APA. This would be the case for a dense sampling of the q‐space, or at least for a truly sparse one. However, actual samplings comprise a structured, regular grid of gradient directions describing a reduced number of shells (b‐values). This way, the measured radial information does not suffice to describe the behavior of the attenuation signal in detail, so that a strong regularization of the prior model is required, leading to a heavily low‐pass filtered estimation of the true EAP. As we report in the results with clinical data (see the PPD experiment), this issue may cause the original PA to lack the expected discriminant power, or even to have less discriminant power than conventional DT‐MRI.

Moreover, Figure 2 suggests that the lack of a proper radial description of the diffusion signal, and the consequent over‐regularization of the problem, may cause EAP estimators like MAP‐MRI to completely blur out white matter regions such as the thalamus or the caudate, which are more clearly defined by the APA.

The experiments carried out in this paper confirm that the proposed measures show a discriminant power that is superior to traditional DT‐MRI markers and, in some occasions, even over the PA. We are aware that the finding of more significant differences between groups does not directly imply that one method is better than other. However, under the assumption that the group differences represented here are true positives (which is endorsed by the related literature), the proposed APA may be reasonably attributed a higher sensitivity.

The main advantage of the proposed measures, when compared to the PA, is that they can be calculated from a reduced set of measures leading to a significant reduction in data acquisition time. Initially, they are intended for data collected with one shell (b‐value), but the methodology can be easily extrapolated to more than one. In addition, the experiments with different gradient directions carried out over the CBR dataset have shown a robustness to differences in the number of gradient directions, which will allow a further reduction in the amount of requisite data, making it compatible with contemporary acquisition protocols widely deployed in studies, with as few as 64 gradient directions. It is a common practice to acquire two shells (eg, $b\;=\;[1000,\;3000]\;s/{\mathrm{mm}}^{2}$) to estimate classical DT‐MRI parameters, like the FA and MD, and advanced models (DKI, HARDI, CHARMED, etc.). The APA (or the DiA) proposed here can also be calculated with no additional effort and without changing the acquisition protocol.

Moreover, since the computation of the APA avoids the estimation of the actual EAP, it can be done in a fast and robust way, that is, without imposing a computational burden to the standard protocols. A whole volume can be processed in a matter of seconds while the processing of the original PA usually takes hundreds of minutes, which obviously limits its applicability.

On the other hand, the major drawback of the APA is the explicit assumption of a specific radial behavior for the diffusion, which cannot characterize the whole q‐space. As a consequence, the selection of the b‐value may impact the absolute values of the measures and difficult multicenter studies. However, we have shown that the relative anatomical differences between different regions are preserved regardless of the absolute changes in APA values: as long as the same b‐value is preserved across each study, the results of different clinical trials in terms of increased/decreased anisotropy should be broadly compared. This is by no means something new to diffusion imaging: it is well‐known that a change in the acquisition parameters (number of gradients, b‐value, resolution, scanner vendor, etc.) seriously affects scalar measures like the FA or the MD.^24^, ^35^

## 6 CONCLUSIONS

The newly introduced APA (or, alternatively, the DiA) can be easily integrated into the processing pipeline of currently existing single‐shell dMRI protocols and databases to unveil anatomical details that remain hidden in traditional FA‐based studies. Its simplicity (it is mainly based on linear fitting of SH coefficients) prevents the need for cumbersome parameter tuning procedures via cross‐validation or trial and error, so that the same setting‐up will suit virtually any acquisition protocol out‐of‐the‐box, regardless of the number of acquired gradients and/or b‐values.

In the case of multi‐shell protocols, and whenever the accuracy in the computation of the full PA gets compromised by the lack of a detailed sampling of the whole q‐space, the proposed measures are a robust and useful alternative.

## Supporting information


**FIGURE S1** Visual comparison of the APA calculated with the two approaches, full (F‐APA) and simplified (APA), together with the absolute error. Slices 42, 52 and 65 of MGH1007 volume from the HCP are used. Both measures have been calculated using using $b\;=\;3000\;s/{\mathrm{mm}}^{2}$
**TABLE S1** Average absolute error between F‐APA and APA for the whole MGH1007 volume. Two different shells are considered
**TABLE S2** Estimated execution time for the calculation of APA and F‐APA for the MGH1007 volume

## Data Availability

The full implementation of AMURA, including the APA and the DiA as described here, may be downloaded for Matlab© and Octave, together with use‐case examples and test data, from: http://www.lpi.tel.uva.es/AMURA.
