# Supplementary material for: Apparent propagator anisotropy from single‐shell diffusion MRI acquisitions
Source: Magn Reson Med. 2020 Dec 13;85(5):2869–81. doi: 10.1002/mrm.28620 (PMC8103173; doi:10.1002/mrm.28620)
Supplement: Supplementary file 1 — FIGURE S1 Visual comparison of the APA calculated with the two approaches, full (F‐APA) and simplified (APA), together with the absolute error. Slices 42, 52 and 65 of MGH1007 volume from the HCP are used. Both measures have been calculated using using b=3000s/mm2 TABLE S1 Average absolute error between F‐APA and APA for the whole MGH1007 volume. Two different shells are considered TABLE S2 Estimated execution time for the calculation of APA and F‐APA for the MGH1007 volume [file MRM-85-2869-s001.pdf]

Supporting Information:

Isotropic equivalent signals under the mono-exponential model

Santiago Aja-Fernández*†, Antonio Tristán-Vega†, and Derek K. Jones‡

†*Laboratorio de Procesado de Imagen (LPI), Universidad de Valladolid, Valladolid, Spain*
‡*Cardiff University Brain Research Imaging Centre (CUBRIC), School of Psychology, University of Cardiff, UK*

September 23, 2020

This document is supplementary material to the document entitled **Apparent Propagator Anisotropy from Single-Shell Diffusion MRI Acquisitions**, by the same authors.

1 Isotropic Ensamble Average Propagator

In the main document, section 3.1, we explicitly calculate the inner product that defines the PA by using the simplification in eq. (9), yielding an anisotropy metric related to the PA for a specific shell, namely the APA. After [1], this metric requires the definition of an isotropic signal equivalent to the mono-exponential model. The rationale behind [1] is that the EAP can be averaged over the directional coordinates to obtain the closest isotropic signal to the original one. By using

$$P(\mathbf{R}) = \int_{\mathbb{R}^3} E(\mathbf{q}) e^{-2\pi j \mathbf{q}\cdot\mathbf{R}} d\mathbf{q} = \mathfrak{F}\left\{|E(\mathbf{q})|\right\}(\mathbf{R}), \tag{1}$$

we have:

$$P_I(\mathbf{R}) \triangleq \frac{1}{4\pi}\int_S P(\mathbf{R}) d\mathbf{r} = \frac{1}{4\pi}\int_S \left(\int_{\mathbb{R}^3} E(\mathbf{q}) e^{-2\pi j \mathbf{q}\cdot\mathbf{R}} d\mathbf{q}\right) d\mathbf{r}, \tag{2}$$

where $\|\mathbf{r}\| = 1$ and $\mathbf{R} = R\mathbf{r}$. The inner integral in $\mathbb{R}^3$ is usually computed in spherical coordinates, so that $d\mathbf{q} = q^2 d\mathbf{u}\, dq$ and a straightforward manipulation yields:

$$\begin{aligned} P_I(\mathbf{R}) &= \frac{1}{4\pi}\int_0^\infty \int_S q^2 E(\mathbf{q}) \left(\int_S e^{-2\pi j \mathbf{q}\cdot\mathbf{R}} d\mathbf{r}\right) d\mathbf{u}\, dq = \frac{1}{2}\int_0^\infty \int_S q^2 E(\mathbf{q}) \frac{J_{1/2}(2\pi qR)}{\sqrt{qR}} d\mathbf{u}\, dq \\ &= \frac{1}{2\sqrt{R}} \int_0^\infty q^{3/2} J_{1/2}(2\pi qR) \left(\int_S E(\mathbf{q}) d\mathbf{u}\right) d\mathbf{q}, \end{aligned} \tag{3}$$

where $J_{1/2}$ stands for Bessel's function of the first kind with index 1/2. The meaning of eq. (3) is that the isotropic EAP defined as the directional average of the original EAP becomes the Bessel

*Corresponging author: sanaja@tel.uva.es.

transform of the isotropic diffusion signal defined as the directional average of the original diffusion signal. In other words, the isotropic equivalent to the EAP corresponds to a diffusion signal that is indeed computed in the same manner:

$$P_I(\mathbf{R}) = \frac{1}{4\pi}\int_S P(\mathbf{R})d\mathbf{r} \quad \longleftrightarrow \quad E_I(\mathbf{q}) = \frac{1}{4\pi}\int_S E(\mathbf{u})d\mathbf{u}. \tag{4}$$

By assuming a mono-exponential model of $E(\mathbf{q})$ itself, we can compute:

- The (squared) ℓ_2 norm of $E(\mathbf{q})$:

$$\begin{aligned}\|E\|^2 &= \int_0^\infty\int_S q^2\left|\exp\left(-4\pi^2\tau q^2 D(\mathbf{u})\right)\right|^2 d\mathbf{u}\ dq = \int_S\int_0^\infty q^2\exp\left(-8\pi^2\tau q^2 D(\mathbf{u})\right)dq\ d\mathbf{u}\\ &= \int_S \frac{\sqrt{\pi}}{4\left(8\pi^2\tau D(\mathbf{u})\right)^{3/2}}d\mathbf{u}.\end{aligned} \tag{5}$$

- The (squared) ℓ_2 norm of $E_I(\mathbf{q})$:

$$\begin{aligned}\|E_I\|^2 &= \int_0^\infty\int_S q^2\left|\frac{1}{4\pi}\int_S\exp\left(-4\pi^2\tau q^2 D(\mathbf{v})\right)d\mathbf{v}\right|^2 d\mathbf{u}\ dq\\ &= 4\pi\int_0^\infty q^2\left|\frac{1}{4\pi}\int_S\exp\left(-4\pi^2\tau q^2 D(\mathbf{v})\right)d\mathbf{v}\right|^2 dq\\ &= \frac{1}{4\pi}\int_0^\infty q^2\left(\int_S\exp\left(-4\pi^2\tau q^2 D(\mathbf{u})\right)d\mathbf{u}\right)\left(\int_S\exp\left(-4\pi^2\tau q^2 D(\mathbf{v})\right)d\mathbf{v}\right)\ dq\\ &= \frac{1}{4\pi}\int_S\int_S\left(\int_0^\infty q^2\exp\left(-4\pi^2\tau q^2\left(D(\mathbf{u})+D(\mathbf{v})\right)\right)\right)d\mathbf{u}\ d\mathbf{v}\\ &= \frac{1}{4\pi}\int_S\int_S\frac{\sqrt{\pi}}{4\left(4\pi^2\tau\left(D(\mathbf{u})+D(\mathbf{v})\right)\right)^{3/2}}d\mathbf{u}\ d\mathbf{v}.\end{aligned} \tag{6}$$

- The inner product between $E(\mathbf{q})$ and $E_I(\mathbf{q})$:

$$\begin{aligned}\langle E, E_I\rangle &= \int_0^\infty\int_S q^2\left[\exp\left(-4\pi^2\tau q^2 D(\mathbf{u})\right)\left(\frac{1}{4\pi}\int_S\exp\left(-4\pi^2\tau q^2 D(\mathbf{v})\right)d\mathbf{v}\right)\right]d\mathbf{u}\ dq\\ &= \frac{1}{4\pi}\int_S\int_S\left(\int_0^\infty q^2\exp\left(-4\pi^2\tau q^2\left(D(\mathbf{u})+D(\mathbf{v})\right)\right)\right)d\mathbf{u}\ d\mathbf{v}\\ &= \frac{1}{4\pi}\int_S\int_S\frac{\sqrt{\pi}}{4\left(4\pi^2\tau\left(D(\mathbf{u})+D(\mathbf{v})\right)\right)^{3/2}}d\mathbf{u}\ d\mathbf{v} = \|E_I\|^2.\end{aligned} \tag{7}$$

- The cosine between both two signals, which implicitly defines the PA:

$$\cos^2\left(\angle[E, E_I]\right) = \cos^2\theta_{E,E_I} = \frac{\int_S\int_S\left(D(\mathbf{u})+D(\mathbf{v})\right)^{-3/2}d\mathbf{u}\ d\mathbf{v}}{4\pi\int_S\left(2D(\mathbf{u})\right)^{-3/2}d\mathbf{u}}. \tag{8}$$

Using this complete approach, we achieve a a closed form of the APA that involves the computation of a quadratic form on the measured values at each voxel. Pursuing an analogous formulation

to that in AMURA [2], in the main document we propose an alternative formulation leading to a linear computation with negligible deviations from the model. To that end, instead of using eq. (4) for the isotropic equivalent, we use the following expression instead::

$$E_I(\mathbf{q}) \triangleq \exp\left(-4\pi^2\tau q^2\ D_{\mathrm{AV}}\right), \tag{9}$$

with

$$D_{\mathrm{AV}} = \frac{1}{4\pi}\int_S D(\mathbf{u})d\mathbf{u}. \tag{10}$$

2 Practical implementations

Note that the computation of $\|E_I\|^2 = \langle E, E_I\rangle$ in eq. (4) requires evaluating a double surface integral in the orientation variables $\mathbf{u}$ and $\mathbf{v}$. In a practical implementation, such integrals are computed based on spherical harmonics expansions. In precise terms, let $\{\mathbf{u}_n\}_{n=1}^N$ be the set of the N acquired gradients within the measured shell; let $\{Y_j(\mathbf{u})\}_{j=1}^M$ be the set of the M first (low order) spherical harmonics (typically $M < N$). The coefficients $\{c_j\}_{j=1}^M$ of a given orientation function, $S(\mathbf{u})$, in this basis will be fitted as a Laplacian-regularized least squares problem:

$$\mathbf{S} \simeq \mathbf{BC} \Rightarrow \mathbf{C} = \left(\mathbf{B}^T\mathbf{B} + \lambda\mathbf{L}^2\right)^{-1}\mathbf{B}^T\mathbf{S}, \tag{11}$$

where the $M \times 1$ vector $\mathbf{C}$ stacks the coefficients c_j; the $N \times 1$ vector $\mathbf{S}$ stacks the measurements of the orientation function, $S(\mathbf{u}_n)$; the $N \times M$ matrix $\mathbf{B}$ stacks the values of the basis functions, $\mathbf{B}_{n,j} = Y_j(\mathbf{u}_n)$; λ is the Laplace-Beltrami regularization parameter so that the $M \times M$ matrix $\mathbf{L}$ contains the eigenvalues of spherical harmonics for the Laplacian (we fix it to constant value of 0.006 in all cases). Since the $0-$th order spherical harmonic encodes the DC component of the signal, the integral of the orientation function over the unit sphere reduces to a scaled version of its c_0 coefficient. For example, eq. (5) can be estimated as:

$$\|E\|^2 = \frac{\sqrt{\pi}}{4\left(8\pi^2\tau\right)^{3/2}}\ \sqrt{4\pi}\ \mathbf{s}\bar{\mathbf{D}}, \tag{12}$$

where $\mathbf{s}$ stands for the first row of $\left(\mathbf{B}^T\mathbf{B} + \lambda\mathbf{L}^2\right)^{-1}\mathbf{B}^T$ and the $N \times 1$ vector $\bar{\mathbf{D}}$ stacks the N values of $D(\mathbf{u}_n)^{-3/2}$. In order to compute eqs. (6) and (7), we arrange a $N \times N$ matrix $\bar{\bar{\mathbf{D}}}$ whose entries are $\bar{\bar{\mathbf{D}}}_{n_1,n_2} = (D(\mathbf{u}_{n_1}) + D(\mathbf{u}_{n_2}))^{-3/2}$. Then:

$$\|E_I\|^2 = \langle E, E_I\rangle = \frac{\sqrt{\pi}}{16\pi\left(4\pi^2\tau\right)^{3/2}}\ 4\pi\ \mathbf{s}\bar{\bar{\mathbf{D}}}\mathbf{s}^T, \tag{13}$$

where the left product with $\mathbf{s}$ stands for the outermost integral in eq. (6) in the variable $\mathbf{v}$ for each constant $\mathbf{u}$ (each column $\mathbf{u}_{n_2}$), meanwhile the right product with $\mathbf{s}^T$ stands for the innermost integral in eq. (6) in the variable $\mathbf{u}$ for each constant $\mathbf{v}$ (each row $\mathbf{u}_{n_1}$). This way, the computational complexity of computing simple surface integrals remains linear with the number of sampled gradients, $\mathcal{O}(N)$, while the complexity of double surface integrals becomes $\mathcal{O}(N^2)$.

3 Comparison of both APA implementations

[Figure 1 about here.]

In order to compare the results given by the simplified APA implementation proposed in the main document to the complete implementation described in this supplementary material, an experiment is carried out. For the sake of comparison, we will denote full-APA (F-APA) to the complete implementation derived from eq. (8), and simply APA to the fast approach used along the paper, derived from eq. (9). For a visual comparison we use three slices from the HCP volume MGH1007 using a single shell for b=3000 s/mm^2. We consider the absolute error as the quality measure:

$$\text{Error}(\mathbf{x}) = |\text{APA}(\mathbf{x}) - \text{F-APA}(\mathbf{x})|.$$

A mask is used in order to limit the measurement of the error to the white matter area. Both measures are bounded in the interval [0,1] and so will be the error. A visual comparison for slices (42, 52, 65) is shown in Fig. S1. The average error for the white matter is calculated for the whole volume at b=3000 s/mm^2 and b=5000 s/mm^2 and it can be found in Table S1. Note that the average error is smaller than 1% for both shells, which implies that both measures are practically the same and both provide very similar results. In addition, from the visual comparison in Fig. S1, we can also conclude that the error is uniformly distributed in the white matter, which, from a practical view point, it can be seen as a very small bias in the measure. Some of the experiments with real data from the main document were also redone for F-APA with no noticeable differences from those with APA.

[Table 1 about here.]

Although both methods provide similar results, in this work we have opted for APA, for a matter of simplicity. As previously stated, the computational complexity of computing simple surface integrals is $\mathcal{O}(N)$, while the complexity of double surface integrals becomes $\mathcal{O}(N^2)$. Since APA is only based on simple integrals, similar results can be obtained in reduced computation time. In order to test it on real data, we measure the execution times of computing the metrics over the MGH1007 volume previously described. The experiment is run on a quad-core Intel(R) Core(TM) i7-4770K 3.50GHz processor under Ubuntu Linux 16.04 So using one single shell in MATLAB R2013b without multi-threading. The results are reported in Table S2. As a consequence of the simplicity on the implementation of APA, it shows execution times 200 faster than F-APA.

Thus, since the implementation error is so small and the gain in execution time is so high, we have opted to use the fast implementation thorough the whole paper.

[Table 2 about here.]

References

[1] Özarslan E, Koay CG, Shepherd TM, Komlosh ME, Irfanoğlu MO, Pierpaoli C, Basser PJ. Mean apparent propagator (MAP) MRI: a novel diffusion imaging method for mapping tissue microstructure. NeuroImage 2013;78:16–32.

[2] Aja-Fernández S, de Luis-García R, Afzali M, Molendowska M, Pieciak T, Tristán-Vega A. Micro-structure diffusion scalar measures from reduced MRI acquisitions. PLOS-ONE 2019;.

List of Figures

S1 Visual comparison of the APA calculated with the two approaches, full (F-APA) and simplified (APA), together with the absolute error. Slices 42, 52 and 65 of MGH1007 volume from the HCP are used. Both measures have been calculated using using b=3000 s/mm^2. 6

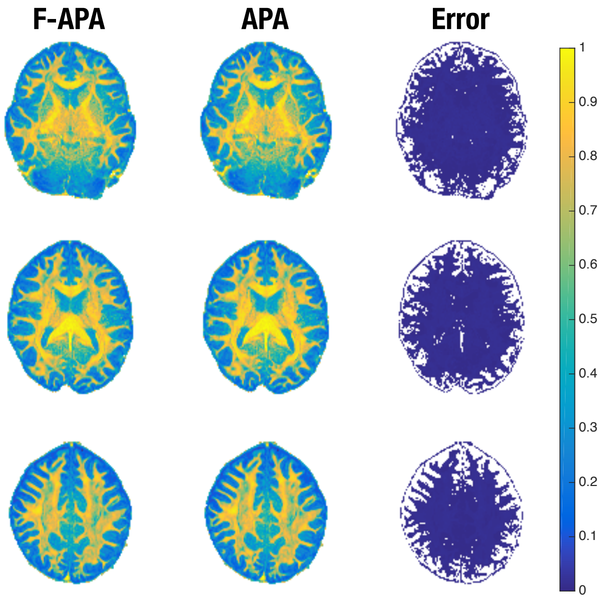

Figure S1: Visual comparison of the APA calculated with the two approaches, full (F-APA) and simplified (APA), together with the absolute error. Slices 42, 52 and 65 of MGH1007 volume from the HCP are used. Both measures have been calculated using using b=3000 s/mm^2.

List of Tables

S1 Average absolute error between F-APA and APA for the whole MGH1007 volume. Two different shells are considered. 8
S2 Estimated execution time for the calculation of APA and F-APA for the MGH1007 volume. 9

Shell (s/mm^2)	3000	5000
Average error	0.0083	0.0083

Table S1: Average absolute error between F-APA and APA for the whole MGH1007 volume. Two different shells are considered.

Shell (s/mm^2)	3000	5000
APA	6.05 s	12.24 s
F-APA	1152 s	2738.75s

Table S2: Estimated execution time for the calculation of APA and F-APA for the MGH1007 volume.
